# One-channel double stent implantation for hilar biliary obstructions

**DOI:** 10.3892/etm.2013.921

**Published:** 2013-01-23

**Authors:** GUANGSHAO CAO, HUICUN CAO, JIAN LIU, YUDAN WANG, ZHENYU WANG

**Affiliations:** Interventional Department, Henan Provincial People’s Hospital, Zhengzhou, Henan 450003, P.R. China

**Keywords:** biliary obstruction, single channel, double stents, antitumor

## Abstract

The aim of this study was to evaluate the effect of percutaneous one-channel double stent implantation on hilar biliary obstruction involving both hepatic ducts and its clinical value. A total of 8 patients with hilar biliary obstruction involving the left and right hepatic ducts were enrolled. A percutaneous unilateral approach was adopted. Two stents were implanted, one between the left and right hepatic ducts and the other between the hepatic ducts and the common bile duct for biliary drainage. Interventional therapies such as arterial chemoembolization were performed for antitumor treatment. All surgical procedures were successfully accomplished. At 2 weeks after stenting, total bilirubin decreased to 61.2±13.4 μmol/l (the preoperative value was 267.1±154.7 *μ*mol/l). No severe complications or mortalities occurred. Single-channel double stent implantation should be the preferred method of treatment for patients with hilar biliary obstruction involving both hepatic ducts. Drainage and antitumor treatment should also be used when necessary.

## Introduction

Malignant bile duct obstruction may develop into clinically serious diseases, including obstructive jaundice and cholangitis. Malignant bile duct obstruction is a condition that is difficult to treat and has a high mortality rate. Secondary hepatic inadequacy as well as digestion and absorption hypofunction following bile duct obstruction-induced cholestasis are the primary causes of accelerated mortalities due to obstructive jaundice. Malignant hilar obstructive jaundice has long been a challenge in clinical practice due to a low resective rate, insufficient drainage and numerous complications. In the treatment of biliary obstructive jaundice, endoscopic retrograde cholangio-pancreatography (ERCP) combined with endoscopic biliary stenting has been extensively applied as a minimally invasive palliative therapy (1,2). Currently, percutaneous biliary stent implantation, due to a higher success rate, the ability to meet physiological requirements and a negligible influence on the quality of life, has become another minimally invasive palliative therapy for malignant hilar obstructive jaundice. In addition, under conditions of unsuccessful surgery or drainage insufficiency, this technique has gradually become an alternative to failed biliary stent implantation under ERCP in 33 to 88% of cases (3–10). One-channel double stent implantation has the advantages of little trauma, few complications and sufficient drainage, and has been increasingly widely used for the treatment of partial hilar biliary obstruction. However, studies on this therapy, particularly its combined use with antitumor interventional techniques, such as arterial chemoembolization, are rare.

Between September 2004 and September 2009, a total of 8 patients with hilar obstruction were subjected to percutaneous one-channel double stent implantation combined with chemoembolization or I^125^ seed implantation for antitumor treatment at the Interventional Department, Henan Provincial People’s Hospital (Zhengzhou, China). The aims of the study were to evaluate the effect, safety and clinical value of percutaneous one-channel double stent implantation combined with anti-tumor treatment.

## Materials and methods

### Clinical data

A total of 8 patients with hilar biliary obstruction, including 5 males and 3 females, were enrolled in the present study. Their ages ranged from 31 to 78 years with an average of 50.2±11.4 years. Among these patients, four had bile duct cancer, two had hilar hepatic carcinoma, one had metastatic carcinoma and one had pancreatic head carcinoma. Their clinical manifestations included jaundice, itching and rock-like stools. Preoperative computed tomography (CT) or magnetic resonance imaging (MRI) revealed hilar lesions which caused bilateral hepatic duct and/or common hepatic duct obstruction but without surgical indications. The surgical instruments used included 18–22 G trocars, balloons, nitinol mesh biliary stents with diameters of 8–10 mm and lengths of 50–80 mm (Nanjing Micro-tech Co., Ltd, Nanjing, China), 7–9 F stent conveyors and 7–8.5 F biliary drainage tubes (Bard Co., Murray Hill, NJ, USA). The study was conducted in accordance with the Declaration of Helsinki and with approval from the Ethics Committee of Henan Provincial People’s Hospital. Written informed consent was obtained from all patients.

### One-channel double stent implantation

**Figure 1:**
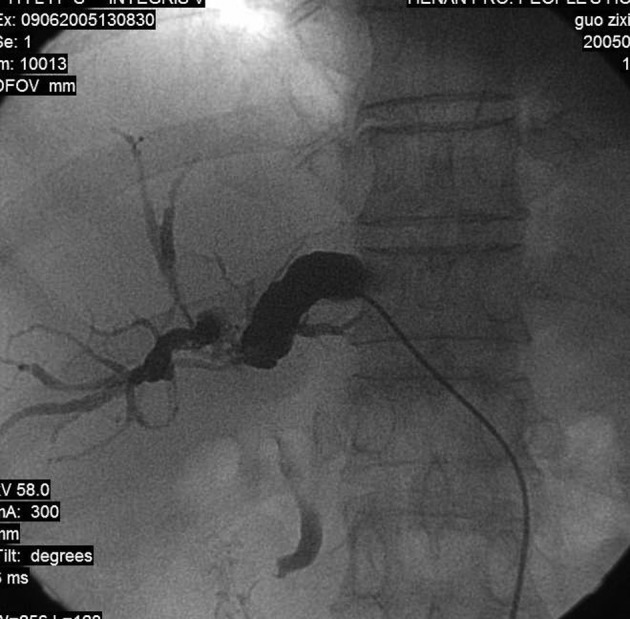
A male 57-year-old patient exhibited an obstruction at the confluence of the left and right bile ducts and clear left bile duct dilation by percutaneous cholangiography.

**Figure 2:**
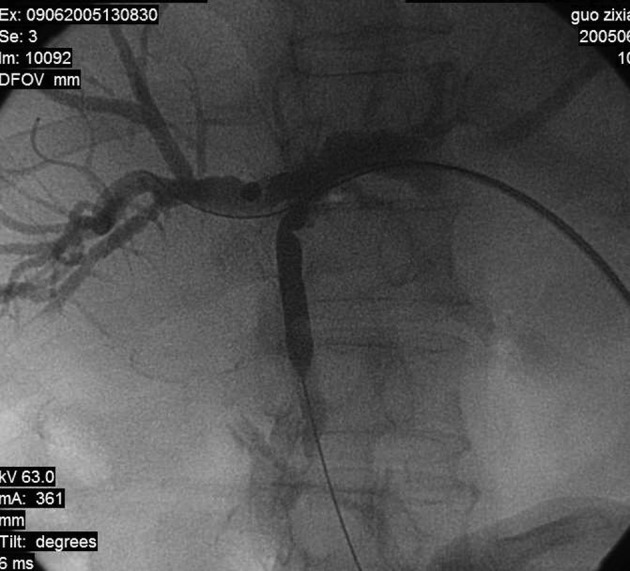
A left approach slightly deviated from the subxiphoid was adopted and the obstructed bile duct segment was expanded by the balloon introduced by a guidewire.

**Figure 3:**
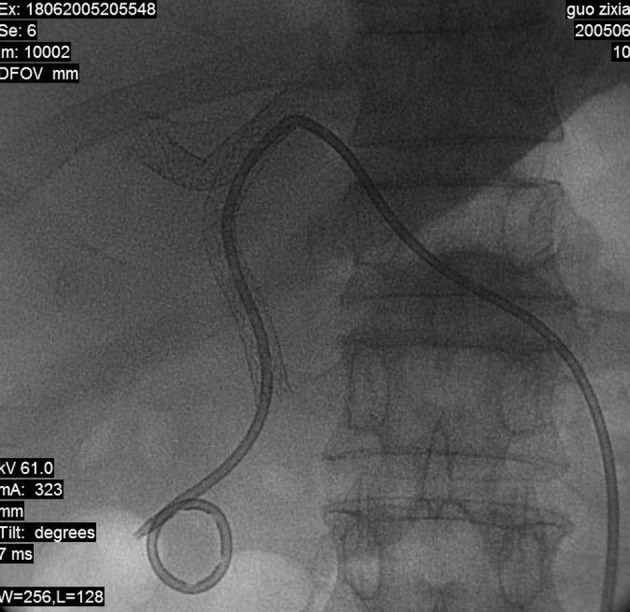
Two stents were implanted between the left and right hepatic ducts, and between the left hepatic duct and the common bile duct in a ‘7’-shaped arrangement.

**Figure 4:**
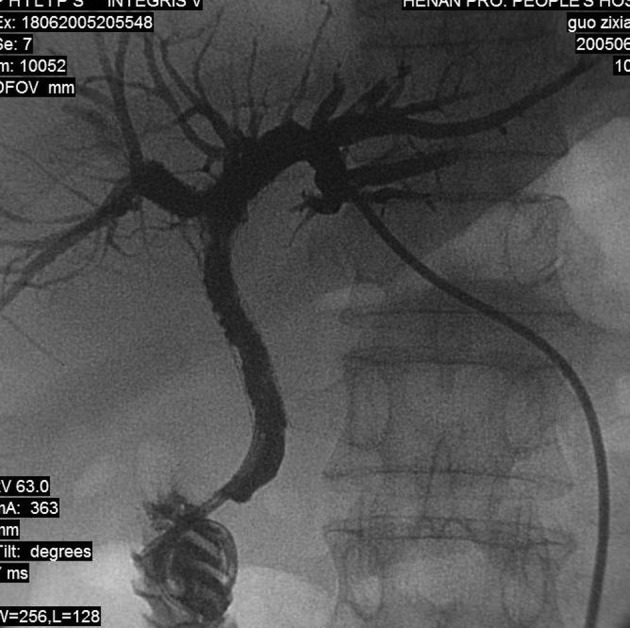
An angiographic review showed that the contrast agent passed through the two stents smoothly.

Surgery was performed under fluoroscopic monitoring. The right hepatic duct was punctured through the right midaxillary line. The route of the biliary system was observed by angiography. A guidewire was inserted through the right hepatic duct and the potential lacune of the obstruction segment between the left and right hepatic ducts was sought. The guidewire entered the left hepatic duct through the obstruction segment in the right hepatic duct. A stent was implanted at the obstruction segment to allow the bile from the left hepatic duct to flow into the right hepatic duct. The guidewire was then sent into the common bile duct through the right hepatic duct and passed down to the duodenum. Another stent was implanted between the right hepatic duct and the common bile duct. Both stents were released after the obstructed segment was sufficiently dilated by a balloon catheter. The two stents were in a reversed ‘7’-shaped arrangement. However, if the right approach was not suitable or the patient showed more noticeable dilation of the left hepatic duct, a left approach slightly deviated from the subxiphoid was adopted. The remainder of the procedure was as described previously, with two stents in a ‘7’ arrangement (Figs. 1–4). If the patient was weak and had numerous diseases, or the obstruction was so severe that it was difficult for the guidewire to pass, the guidewire was adjusted as much as possible to enter the opposite intrahepatic bile duct. An external biliary drainage tube was introduced into the opposite bile duct along the guidewire for simultaneous external drainage of the intrahepatic bile ducts. Stent implantation was performed when jaundice improved 3–10 days later.

Following implantation, stent morphology and locations were observed via transcatheter angiography. The puncture tract was sealed off with a gelatine sponge. Under conditions of bile duct hemorrhage, incomplete stent opening or preoperative diagnosis of cholangitis, external biliary drainage was maintained. At 3–7 days, a follow-up angiography was performed. When a contrast agent was confirmed to enter the duodenum smoothly, the drainage tube was removed.

### Combined antitumor treatment

Percutaneous I^125^ seed implantation into cancer with insufficient blood supply was performed for six patients, including 4 patients with bile duct cancer, one patient with metastatic carcinoma and one patient with pancreatic head carcinoma, and chemoembolization was performed for hepatocarcinoma with a rich blood supply was performed for two patients. The commonly used drugs were pirarubicin (40 mg), mitomycin (20 mg) and embolization agents (between 10 and 20 ml). The time between interventions was approximately 1 month over a course of 1–3 treatments.

## Results

### Surgery and postoperative effect

A total of 16 stents were successfully implanted into 8 patients. A right approach was adopted for 6 patients, with the drainage between stents in a reversed ‘7’ shape. A left approach was adopted for 2 patients, with the drainage between stents in a ‘7’ shape. Immediate stent implantation following puncture was performed for 7 patients. One patient received early external drainage of both hepatic ducts and the biliary duct stents following the improvement of jaundice. Immediate postoperative sealing of the puncture tract was implemented in 7 patients. The external biliary drainage of one patient was arrested due to a small amount of bile duct hemorrhage. At 3 days, the patient’s drainage fluid turned yellowish and an angiographic review showed satisfactory stent locations and morphology, as well as smooth bile duct drainage. The external drainage tube was then extracted. The preoperative total bilirubin concentrations in the patients ranged from 138.2 to 796.9 *μ*mol/l with an average of 267.1±154.7 *μ*mol/l. This range decreased to 40.1–256.3 *μ*mol/l with an average of 61.2±13.4 *μ*mol/l at 2 weeks after surgery. The patients were followed up for 1–12 months. One mortality occurred within 1 month and five mortalities within 2–6 months. Two patients survived for >6 months.

### Complication management

No severe complications or mortalities associated with the implanted stents occurred. One patient exhibited a small volume of bile duct hemorrhage but had stable vital signs. Following external drainage and the application of hemostatics, hemorrhaging stopped 2 days later. Another patient showed persistent low-grade fever (∼38°C) but without rigors or convulsions. The fever improved following an anti-infective therapy. The long-term complications in this study mainly took the form of in-stent restenosis. During follow-ups, one patient exhibited in-stent restenosis/obstruction. After external drainage, the jaundice improved.

## Discussion

Hilar biliary obstruction refers to a medical condition in which primary bile duct carcinoma at the confluence of the liver, common bile duct and both hepatic ducts, or other malignancies near the site, encroaches or compresses the bile ducts, causing obstruction at any site of the bile ducts, particularly bile duct carcinoma jaundice. Hilar biliary obstruction does not have specific clinical manifestations. When diagnosed, the majority of patients have already progressed to late stage disease and have therefore missed the optimal surgical opportunity. In addition, the *porta hepatis* has a complicated anatomical position, which contributes to a 5-year survival rate of <10% and a resective rate of <20%, with >80% of patients receiving palliative treatment (11). The biliary-intestinal anastomosis and ‘T’ tube drainage commonly used in surgery may cause extensive trauma and are likely to induce restenosis or obstruction. As endoscopic and interventional techniques develop, biliary stent drainage under ERCP and percutaneous transhepatic choleductus drainage have gradually replaced the previous surgical methods (3,12). Endoscopic and percutaneous methods of biliary decompression offer relief from malignant biliary obstruction, and each method has its own performance characteristics depending on the obstruction site (13). Compared with ERCP, percutaneous transhepatic choleductus drainage is safer, more effective and advantageous to survival due to the applications of ultrasound-guided devices and self-expandable metallic stents (1–10). However, patients with Klatskin tumors or malignant hilar obstruction do not tolerate drainage failure well (13). In addition, a significant increase has been observed in the survival of patients with initial successful drainage compared with those having a failed first attempt but subsequent success (8.7 months vs. 1.8 months; P<0.001) (14).

Previously, interventional therapies focused only on dominant bile duct single-stent drainage. However, partial drainage, particularly the failure of drainage of the bile duct obstruction that may be observed by percutaneous transhepatic chlangiography (15), causes liver function damage to the undrained bile duct area and may lead to cholangitis and hepatophyma (16), thus further influencing the antitumor treatment efficacy and patient prognosis. Deviere *et al* reported the serious negative impact on patient outcomes of sepsis in undrained segments (17). Life-threatening septic complications may occur and prolonged sepsis may delay or even disqualify patients from the intended treatment. Therefore, effective bile duct drainage not only improves quality of life and extends survival but also provides an opportunity for radiotherapy, chemotherapy or even radical surgery for certain patients (1,18–20). Multi-stent implantation may enlarge drained segments and thus achieve comprehensive and complete internal bile drainage of the obstructed bile ducts. If it is possible to establish improved internal drainage in the three main bile ducts (the right anterior, right posterior and left lobes), internal drainage should be attempted where possible. Multi-stent implantation should be adopted to achieve complete internal static bile drainage, particularly in patients with hilar biliary obstruction who are not able to receive surgical treatment.

To date, multi-stent implantation in clinical practice mainly takes the forms of double-channel and one-channel double stent implantation. The former is performed by puncturing bilateral hepatic ducts through the right midaxillary line and the subxiphoid and then implanting two stents for drainage with the stents passing through the stenotic segment and running in the common bile duct in a ‘kissing’ or ‘Y’-shaped arrangement. The latter is performed through a single puncture tract to establish a stent internal drainage channel between the left and right hepatic ducts, and then an internal drainage channel between the right hepatic duct and the common bile duct for complete and sufficient bile drainage, with the two stents in a ‘7’- or reversed ‘7’-shaped arrangement. Compared with the former, the latter requires fewer punctures, thereby alleviating pain and reducing the risks of puncture-associated complications, including hemorrhage, biliary fistula and biliary tract infection. However, it requires a higher level of surgical skill.

Prior to surgery, the dilation of the intrahepatic bile ducts and the included angle between the left and right hepatic ducts shown by CT or MRI should be well known. Based on these observations, the site and direction of puncture are determined. Normally, the midaxillary line is the site for a puncture in the right hepatic duct. However, if such a puncture is not appropriate, or the dilation of the left hepatic duct is greater, a left approach should be adopted in which a puncture in the left hepatic duct is implemented at a site slightly deviated from the subxiphoid. Puncturing should be monitored fluoroscopically. After successful puncturing, angiography is performed, and the direction of needle insertion is adjusted to obtain the appropriate angle. Normally, a second- or third-grade bile duct branch is used as the puncture entry point as it is comparatively distant from the confluence of the left and right hepatic ducts and thus allows greater room for a catheter, as well as a large space for stent or internal drainage tube release at the proximal end, thereby increasing the drainage achievement rate and surgical safety. However, since patients with obstructive jaundice have thin bile duct walls, puncturing may easily damage the opposite bile duct wall. In addition, both an inappropriate direction of the guidwire through the puncture site and an excessive use of force may cause bile duct perforation. Therefore, a ‘J’-shaped super-smooth guidewire should be used. During insertion, the guidewire should be softly twirled with a slightly-strengthened pushing force to avoid false passage, perforation, or even biliary fistula or biloma. After the guidewire passes through the obstructed segment an angiographic catheter is sent along the guidewire. Balloon dilation may be performed after angiography shows that the catheter is located anterior to the bile duct. For patients with a serious obstruction where it is difficult to pass the guidewire through the stenotic segment, 3–10 days of external drainage may be performed. After such a treatment, icteric indices decrease, liver function recovers, constitutional symptoms improve, intra-bile duct pressure and tension reduce and bile duct wall edema regresses. This contributes to the easy passage of the guidewire through an obstructed segment and the recanalization of certain originally obstructed bile ducts. Since a percutaneous transhepatic puncture channel has been established by this time, stent implantation tends to be easier to perform with less hemorrhage or pain.

However, postoperative intra-stent obstruction remains an urgent clinical challenge influencing the middle- and long-term treatment effect. Once stenosis or obstruction recurs, another puncture drainage or stent implantation is required. This phenomenon inevitably increases the surgical difficulty and causes rehospitalization, resulting in further complications and increased medical care costs (21,22). Primary hilar bile duct and pancreatic cancer metastasis or metastatic lymph nodes compressing the *porta hepatis* develop into noninfiltrating hilar carcinoma (23). Under these conditions, the rate of tumor growth inside the lumen of the stent is rather slow. However, for infiltrating hilar carcinoma, including gallbladder carcinoma and hepatocarcinoma, its constant infiltrative growth through the mesh screens of a stent or longitudinal development passing over the top of a stent tends to cause stent obstruction. In addition, as the tumor grows rapidly, intrahepatic metastases encroaching on bile ducts often occur, leading to intrahepatic and extrahepatic malignant strictures. In such conditions, the treatment of primary tumors appears to be critical. Since the tumors are not able to be resected, interventional therapy provides a satisfactory choice. Hepatic arterial chemoembolization has a curative effect on tumors with sufficient blood supply. In the present study, all patients receiving chemoembolization exhibited reduced tumor volumes and improved lipiodol deposition one month after treatment according to CT images. For bile duct carcinoma or metastatic carcinoma with insufficient blood supply, percutaneous I^125^ seed implantation may be applied. Due to the persistent release of radiation, this technique is able to kill tumors, inhibit tumor growth, and extend the duration of unobstructed biliary stents. However, due to the small number of patients receiving such treatment in this study, as well as a lack of a randomized control trial, its curative effect remains unclear.

In summary, to reduce the incidence rates of puncture-associated injury, hemorrhage and infection, single-channel double stent implantation should be considered a priority among interventional therapies for patients with hilar biliary obstruction involving both hepatic ducts. However, since a small included angle between the right and left bile duct and serious obstruction may lead to treatment failure in clinical practice, single-channel or double-channel double stent implantation should be selected according to the morphology and angles of the bile duct branches. For these patients, external drainage and antitumor treatment should also be used when necessary to further improve the comprehensive curative effect. This study may provide a new insight for the treatment of malignant hilar single-duct obstruction in clinical practice. With the continual development of the interventional technique and of interventional equipment, and the possible emergence of new intra-bile duct angle-adjusting puncture instruments and excellent biliary duct stents, improvements in the middle- and long-term curative effects appear to be likely.
